# The End of Volume Five

**Published:** 1890-06

**Authors:** 


### THE END OF VOLUME FIVE.

This issue completes the fifth year of the JOURNAL'S existence. It has prospered, thanks to the generous support of the better class of Texas physicians; and in taking a retrospect, upon the whole, the management has reason to congratulate itself, and nothing to regret. We shall continue to strive by an upright, straightforward and independent course, to merit a continuation of the support, moral and pecuniary, bestowed upon us; and to make the JOURNAL, as a representative of Texas medicine and an exponent of its best phases, a welcome visitor in the office of the active practitioner; to make it a suggestive help to him in his labors, and at the same time a "newspaper" of the profession; to keep the reader posted, not only on medicine and medical practice, but as to what the organized branch or branches are doing, by the publication of their proceedings, and official announcements and papers, and also news affecting the social and professional status of the physicians throughout the State.

We make our bow, and solicit a prompt renewal of subscriptions. If our labors, which have borne good fruit, as witnessed by the extensive organization effected throughout the State, have met your approval, give the JOURNAL the advantage of your support and influence. It will ever strive for the advancement and elevation of legitimate medicine; and having no fears, and asking no favors, it will be as ever, "independent in all things and neutral in nothing" that touches the interest of the respectable portion of the medical profession of the State.

